# An algorithm to identify patients aged 0–3 with rare genetic disorders

**DOI:** 10.1186/s13023-024-03188-9

**Published:** 2024-05-02

**Authors:** Bryn D. Webb, Lisa Y. Lau, Despina Tsevdos, Ryan A. Shewcraft, David Corrigan, Lisong Shi, Seungwoo Lee, Jonathan Tyler, Shilong Li, Zichen Wang, Gustavo Stolovitzky, Lisa Edelmann, Rong Chen, Eric E. Schadt, Li Li

**Affiliations:** 1 Department of Pediatrics, University of Wisconsin School of Medicine and Public Health, Madison, WI USA; 2 GeneDx Holdings Corp, (formerly known as Sema4 Holdings Corp.), Stamford, Connecticut, CT USA; 3 Department of Pediatrics, Icahn School of Medicine at Mount Sinai, New York, NY USA; 4 Department of Genetics and Genomic Sciences, The Icahn Institute for Genomics and Multiscale Biology, Icahn School of Medicine at Mount Sinai, New York, NY USA

**Keywords:** Digital phenotyping, Algorithm, Pediatric genetic disorders, Clinical decision-making

## Abstract

**Background:**

With over 7000 Mendelian disorders, identifying children with a specific rare genetic disorder diagnosis through structured electronic medical record data is challenging given incompleteness of records, inaccurate medical diagnosis coding, as well as heterogeneity in clinical symptoms and procedures for specific disorders. We sought to develop a digital phenotyping algorithm (*PheIndex*) using electronic medical records to identify children aged 0–3 diagnosed with genetic disorders or who present with illness with an increased risk for genetic disorders.

**Results:**

Through expert opinion, we established 13 criteria for the algorithm and derived a score and a classification. The performance of each criterion and the classification were validated by chart review. *PheIndex* identified 1,088 children out of 93,154 live births who may be at an increased risk for genetic disorders. Chart review demonstrated that the algorithm achieved 90% sensitivity, 97% specificity, and 94% accuracy.

**Conclusions:**

The *PheIndex* algorithm can help identify when a rare genetic disorder may be present, alerting providers to consider ordering a diagnostic genetic test and/or referring a patient to a medical geneticist.

**Supplementary Information:**

The online version contains supplementary material available at 10.1186/s13023-024-03188-9.

## Background

The widespread adoption of electronic medical record (EMR) systems has the potential to enable large-scale population-based studies characterizing patients with rare disorders [1]. Groups from Clinical Sequencing Exploratory Research (CSER) and Electronic Medical Records & Genomics (eMERGE) have identified these patient populations by locating genomic information from EMR systems [2, 3]. However, they have also noted that genetic information is most commonly stored in unstructured formats, such as PDF files or in paragraphs of free text in medical notes, making genetic information results challenging to find. Additionally, CSER and eMERGE have not pursued a global approach to identifying patient populations with confirmed genetic disorders or patients yet to be diagnosed with a genetic condition, but rather whose medical records indicate that diagnostic genetic testing is warranted. Indeed, digital phenotyping studies using EMR data have largely focused on identifying populations with specific individual diseases, such as extracting patients with pediatric epilepsy, childhood obesity, or Noonan syndrome [4–7].

When using EMR data to identify patient populations affected with rare genetic disorders, focusing on a specific rare genetic disorder diagnosis for any given patient is error-prone for many reasons. First, of 6519 rare disorders assessed, only 11% have International Classification of Disease 9 (ICD-9) codes and 21% have ICD-10 codes; some ICD codes are nonspecific, often with multiple phenotypes corresponding to a single ICD code [8]. Furthermore, physicians and clinicians sometimes log certain ICD codes as they rule in or out a given condition, or when a condition is part of a differential diagnosis, yet still unconfirmed. Diagnosis codes may also be inaccurate or incomplete [9].

Accordingly, algorithms that assess the risk of genetic disorders have the potential to improve healthcare delivery by assisting physicians and clinicians with clinical decision-making, including guiding when to order a diagnostic genetic test and/or refer a patient to a medical geneticist or other specialists. Further, such algorithms could also be leveraged to identify rare genetic disorders patient populations to carry out cross-sectional and longitudinal epidemiological studies, assess healthcare utilization, and flag patients who may be considered for participation in specialized undiagnosed disease programs and precision medicine initiatives as underdiagnosis of rare genetic disorders is not uncommon [10].

As a collaborative, multidisciplinary team, we developed a digital phenotyping algorithm that used structured EMR data and assessed 13 criteria to identify patients from birth to 3 years of age who have been diagnosed with a rare genetic disorder or who are at high risk for such a diagnosis. We validated the algorithm through blinded chart review by a pediatrician and a clinical geneticist. To the best of our knowledge, we are the first to generate a digital phenotyping algorithm beyond using ICD codes to identify children presenting with illness with an increased risk for genetic disorders and employed this algorithm to assess healthcare outcomes in a large, diverse, pediatric population.

## Results

### Distribution of the 13 criteria in PheIndex

**Fig. 1:**
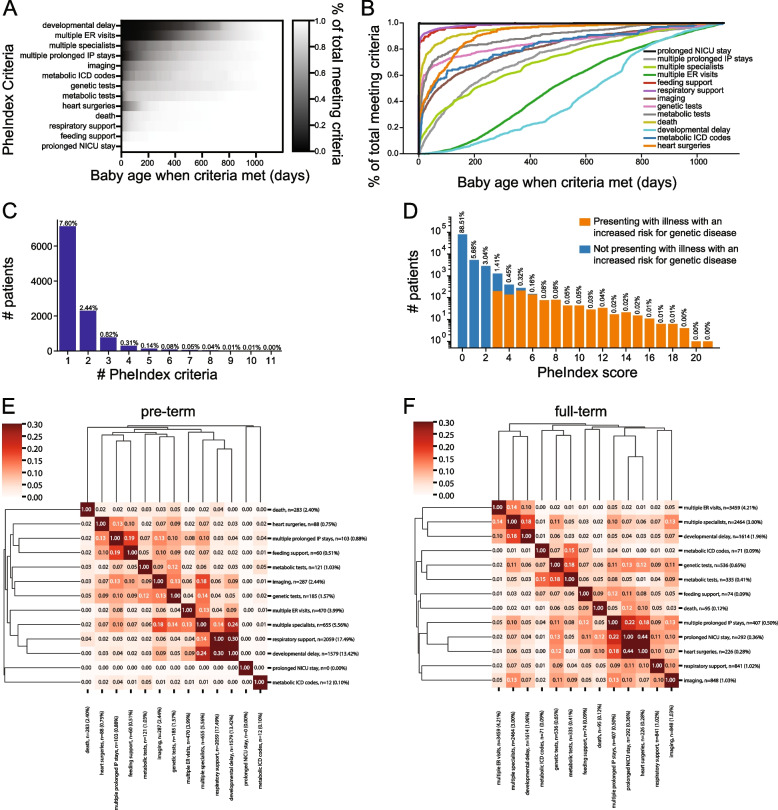
Distribution of *PheIndex* critera of children in the cohort. **A**, **B** Cumulative distribution of time when patients first meet each of the 13 *PheIndex* criteria. Only patients that met each criterion within the three-year limit were included in each cumulative distribution. **A** is sorted by the percentage of patients meeting the criteria at 200 days (least number of patients at the top). **C** Bar graph showing the number and percentage of patients with passing different numbers of *PheIndex* criteria. **D** Distribution of *PheIndex* scores for children within the mother–child cohort. **E**, **F** Clustered heatmap showing the Jaccard index between possible pairs of *PheIndex* criteria in the pre-term (**E**) and full-term (**F**) cohorts. The number and percentage of patients for each criterion are labeled

Our cohort included 93,154 newborns linked to 68,893 mothers who delivered in the Mount Sinai Health System (MSHS) from 2007 to 2019, with clinical features collected to 2020 (Table 1). We first assessed the frequency of each of the 13 *PheIndex* digital phenotyping criteria in our cohort and summarized the number of children aged 0 to 3 years old that satisfied each of the 13 criteria (Supplemental Table S1). The most common criteria were multiple ER visits (3,919; 4.22%), followed by developmental delay (3,159; 3.39%), and multiple visits to specialists (3,091; 3.32%). The least common criteria were metabolic disease diagnosis codes (82; 0.09%) and feeding support (132; 0.14%). Fig. 1A and 1B demonstrate the expected temporal relationship for achieving each criterion.

**Table 1:** Demographic information of the study cohort (*N* = 93,154)

			**All**
		#	*93,154*
Demographics & socioeconomics of mothers		Delivery age, median [Q1,Q3]	32.5 [28.2,36.1]
	Race, n (%)		
		African-American/Black	9423 (10.1)
		Asian	6911 (7.4)
		Caucasian/White	52,667 (56.5)
		Hispanic/Latino	15,543 (16.7)
		Native American	201 (0.2)
		Other	5747 (6.2)
		Unknown	2662 (2.9)
Health Insurance		Mother on Medicaid, n (%)	29,219 (31.4)
		Child on Medicaid, n (%)	27,392 (29.4)
		Child switched to Medicaid, n (%)	154 (0.2)
Birth of children		Year of birth, median [Q1,Q3]	2015 [2011,2017]
		Pre-term birth, n (%)	11,676 (12.5)
	Birth facility, n (%)		
		Mount Sinai Hospital	79,350 (85.2)
		Mount Sinai West	5916 (6.4)
		Other	7888 (8.5)
Record completeness		latest follow-up age (days), median [Q1,Q3]	16.0 [0.0,596.0]
		# of encounters, median [Q1,Q3]	2.00 [1.00,6.00]

We generated a heatmap to show the number and percentage of patients who fell into different major and minor criteria combinations (Supplemental Fig. S1). The distribution for the total number of criteria for each child is given in Fig. 1C. A large majority of patients (88.51%) did not meet any of the 13 criteria, and 98.55% met ≤ 2 criteria. We showed the distribution of *PheIndex Classification* – children who presented with illness with an increased risk for genetic disorders or not – stratified by the *PheIndex Score* (Fig. 1D), as the *PheIndex Classification* depends on the specific combination of major and minor criteria for each patient. The majority of patients had a *PheIndex Score* ≤ 2 (97.23%), indicating that most children in our study population were not likely to have a rare genetic disorder. With our 13 criteria, the *PheIndex Classification* identified 1,088 children who were presenting with illness with an increased risk for genetic disorders out of 93,154 children (1.2%).

In the full-term cohort, *heart surgeries* and *prolonged NICU stay* had the highest Jaccard similarity of 0.44, in line with what we would expect to observe clinically (see Fig. 1E and 1F). In the pre-term cohort, prolonged NICU stay was not chosen to be a criterion because the majority of pre-term infants have an extended NICU stay regardless of whether they have a rare genetic disorder or not. Additionally, *multiple specialists* and *developmental delay* had a Jaccard similarity coefficient of 0.24 (the second highest ranked similarity) in the pre-term cohort and 0.18 (the third highest ranked similarity) in the full-term cohort, consistent with standard clinical practice in which those with developmental delay are referred to specialists such as developmental pediatricians, pediatric neurologists, and/or clinical geneticists.

### Validation of PheIndex: 13 criteria and overall classification

First, we evaluated the accuracy of the values that were extracted from the EMR and assigned to the 13 different criteria for each patient, by comparing *PheIndex’s* identification of each of the 13 criteria against a pediatrician’s evaluation directly from the clinical notes for each patient, for a sample of 200 children (Table 2). The 200 children were sampled from those classified as presenting with illness with an increased risk for genetic disorders positive for a rare genetic condition (*N* = 100) and those classified as negative (*N* = 100). From this comparison, our digital phenotyping algorithm achieved an average accuracy of 94% across the 13 criteria. Accuracies were ≥90% for all criteria except for “*prolonged NICU stays”*, which yielded an accuracy of 81%.

**Table 2:** Accuracy of digital phenotyping algorithm compared to chart review for individual *PheIndex* criteria

PheIndex Criteria	Accuracy
prolonged NICU stay	81%
prolonged inpatient stays	98%
multiple ER visits	94%
multiple specialists	93%
feeding support	96%
respiratory support	90%
imaging	97%
genetic tests	96%
metabolic tests	96%
death	98%
metabolic ICD codes	97%
developmental delay	93%
heart surgeries	97%

Next, we compared the *PheIndex Classification* against the classifications made by a pediatrician/medical geneticist (Table 3). Among the 200 children reviewed, 12 patients did not have sufficient clinical information for the medical geneticist to assess whether a genetic disorder may be present. Ten of these 12 patients were born extremely prematurely (born before 28 weeks gestation), which led to uncertainties as to whether the criteria that were met was because of prematurity or because of an underlying genetic disorder (as determined by the medical geneticist). Therefore, these 12 patients were excluded from this performance evaluation. Among the 188 patients remaining (88 classified as positive by *PheIndex* and 100 classified as negative), 85 patients were deemed to be true positives (definitively or possibly has a rare genetic disorder by chart review, 90% sensitivity/recall) and 91 patients were deemed to be true negatives (does not have a genetic disorder, 97% specificity). Three patients who were classified as positive by *PheIndex* were not thought to have a genetic disorder (false positive), and 9 patients were thought to definitively or possibly have genetic disorders but were classified as negative by *PheIndex* (false negative), yielding a positive predictive value (PPV) of 97%, negative predictive value (NPV) of 91%, and 94% accuracy. If we considered the prevalence of rare genetic disorders to be 3–3.6% of all livebirths [11], the adjusted PPV ranges from 48.1% to 48.3% [12].

**Table 3:** Performance of *PheIndex* classification against chart review

PheIndex Classification	Clinical geneticist classification			
	**Does not have genetic disease**	**Definitively or possibly has genetic disease**	**Unknown (insufficient information)**	Total
Negative	91 (True Negative)	9 (False Negative)	0	100
Positive	3 (False positive)	85 (True Positive)	12	100
Total	94	94	12	200

## Discussion

Identifying pediatric patients across an entire population with or who possibly have a rare genetic disorder is critical for improving patient outcomes. We and others have attempted to identify patients with specific genetic disorders using EMR data, but have found that such a process is not straightforward, largely due to coding differences, unconfirmed diagnoses, variation in disease names and terminology, and inaccurate information represented in medical records [13, 14]. For most rare genetic disorders, it is difficult to identify patients with specific diagnoses, given ICD codes are often nonspecific [1, 15, 16]. Additionally, seeking to analyze individual diseases, even in EMR databases with millions of patients, would result in underpowered studies given the low frequency of individual rare genetic disorders.

In this study, we developed a novel, rule-based digital phenotyping algorithm (*PheIndex*) that utilizes 13 criteria to derive a *PheIndex Score* for children from birth to 3 years of age, in order to classify whether a child is presenting with an illness that may be a rare genetic disorder. Importantly, our score is an evaluation of overall health rather than the presence of specific features of individual diseases. To our knowledge, such an approach has not been developed previously. The criteria for the *PheIndex Score* include items that could be extracted from the EMR with a high degree of precision and accuracy. Our *PheIndex Score* may be utilized for various purposes, including its use as a clinical guide to shorten the diagnostic odyssey of hard-to-diagnose patients, timely administration of therapeutics by facilitating more rapid diagnosis, and/or assessing clinical benefit of genetic testing, all of which help enable the practice of precision medicine in a way that may be more accessible to all. Chart review from clinical genetics experts, confirmed that our *PheIndex* algorithm has the following performance characteristics when the numbers of cases and controls are equal: precision of 97%, recall of 90%, and accuracy of 94%.

### Limitations

While our study population is likely representative of other large, diverse metropolitan areas, it may be less representative of smaller-sized cities and rural areas. While we provided an adjusted PPV of 48% based on an estimated prevalence of rare genetic disorders in the general population, precise estimates of rare genetic disorder prevalence are unavailable, and may also not reflect the PPV for the target population of our algorithm (i.e. children aged 0–3) due to differences in age of onset [11]. Another potential limitation of our study is that we used only de-identified data available in structured EMR databases, and thus did not include all the information that would be available to physicians, such as clinical notes and results of genetic testing. However, despite not having access to all available medical records, our digital phenotype agreed with physician chart review 94% of the time (under conditions in which the number of cases and controls were sampled to be the same), proving that our algorithm successfully identifies children with an increased risk for genetic disorders. In the few occasions where there were discrepancies, this was typically due to incomplete documentation of orders, such as respiratory support and feeding support in *PheIndex* negative children that was uncovered in the notes during chart review. The accuracy of the prolonged NICU stay rule was likely lower than other criterion because an indicator for NICU was not available in the de-identified dataset, and had to be derived by whether an ICU encounter had occurred within a 7-day timeframe from the date of delivery of a child. We also observed that transfers into the NICU were better captured in clinical notes than in the structured data. Nonetheless, the accuracy was still high at 81%, and we intend to build upon this in a future iteration and improvement of the algorithm using the *PheIndex* criteria extracted from notes in addition to structured EMR data.

## Conclusions

In summary, we utilized a comprehensive EMR to develop a novel digital phenotyping algorithm for identification of a pediatric population with a definitive or possible genetic disorder. Our method utilizes a global approach as opposed to identifying patients in the EMR with each specific genetic disorder, which is fraught with misdiagnoses and error. We believe that our *PheIndex* algorithm will address an unmet need to identify children with rare genetic disorders and potentially help overcome well-known obstacles such as underdiagnosis and delayed diagnosis [17].

## Methods

### Construction of mother–child cohort

We obtained de-identified EMR data through June 30, 2020 from the Mount Sinai Health System (MSHS). The newborns in this cohort were born from 2007 to 2019, ensuring that all newborns had at minimum one year of follow-up. To accurately ascertain the gestational age at birth and determine the term status of a newborn, a mother’s EMR had to be linked. In other words, we identified the mother–child pairs, where we obtained mothers’ delivery records for pregnant women who delivered in the MSHS and linked their corresponding newborn with the pregnancy and delivery journey. In total, we identified 93,154 mother–child pairs delivered at MSHS hospitals, covering 68,893 mothers and 93,154 children [18–20]. Moreover, for the children, we obtained gestational age and all diagnoses, procedures, vital signs, laboratory tests, and medications available in the EMR from birth until any subsequent hospital encounters of any type up to three years of age. This study was approved by the Mount Sinai institutional review board (IRB): IRB-20–01771.

### Digital phenotyping algorithm for rare genetic disorders

The general criteria underlying the *PheIndex* (Phenotype Index) digital phenotyping algorithm were established by a clinical geneticist to target children with possible genetic disease based on characteristics often observed in this population. This includes multi-system disease, increased utilization of health care services, more pronounced support, and detailed work-up with laboratory tests and imaging. Therefore, the algorithm comprises criteria primarily based on hospital encounters, procedures, specialist visits, and laboratory test orders. Orders that were subsequently cancelled were not considered. Diagnostic codes of feeding support, developmental delay, and metabolic disease (see Supplemental Table S2A-C), and death, were also used and chosen based on review of a complete list of ICD ontology. A total of thirteen criteria were derived for the algorithm and their description with the associated scores are listed in Table 4. The clinical geneticist received informal input from multiple clinical geneticists and genetic counselors when developing the criteria. Four criteria take into account term status (pre-term or full-term) given that it is expected that pre-term births have higher healthcare utilization on average compared to full-term births.

**Table 4:** Description and scoring for the 13 *PheIndex* criteria

Description	Scoring
*Prolonged stay in the neonatal intensive care unit (NICU) for term babies.* Full-term newborns who were admitted to the NICU and stayed for ≥ 4 days	Major; score = 3
*Prolonged or multiple hospitalizations after discharged from birth.* Hospitalization is defined as an inpatient stay with a duration ≥ 48 h. We included hospital stays where the calculated gestational age is older than 35 weeks and exclude the first newborn encounter if earlier than 35-week gestation. To meet this criterion, the patient must have either at least one prolonged hospitalization (≥ 14 days) or at least two hospitalizations (≥ 48 h duration) for full-term or ≥ 3 hospitalizations (≥ 48 h) for pre-term babies	Major; score = 3
*Visits or consults with multiple specialists other than general pediatricians* Twenty types of specialists were considered: Medical Genetics, Neurosurgery, Pediatric Allergy and Immunology, Pediatric Cardiology, Pediatric Dermatology, Pediatric Endocrinology, Pediatric GI/Pediatric Liver, Pediatric Hematology/Oncology, Pediatric Nephrology, Pediatric Neurology, Pediatric Ophthalmology, Pediatric Orthopedics, Pediatric Otolaryngology, Pediatric Pulmonology, Pediatric Rheumatology, Pediatric Surgery, Pediatric Urology, Transplant, Plastic Surgery. We counted the types of specialists each patient visited or consulted with and not the number of individual specialist visits. Preterm babies with ≥ 4 types of specialists or full-term babies with ≥ 3 types of specialists meet this criterion. We excluded Pediatric Infectious Disease specialty visits as infections in general are primarily due to environmental and non-genetic etiologies, and our aim was to identify a patient population enriched for children with genetic disorders	Minor; score = 1
*Multiple emergency room (ER) visits* Full-term babies with ≥ 5 ER visits or preterm babies with ≥ 7 ER visits meet this criterion	Minor; score = 1
*Feeding support (Gastrostomy tube)* Patients who required feeding support were identified using ICD codes (Supplemental Table 2A) and procedures with description of “nasogastric”, “gastrostomy” and “feed”, or “gastrostomy” and “tube” in the procedure name	Minor; score = 2
*Respiratory support (tracheostomy and mechanical ventilation outside of surgery)* We used tracheostomy and ventilation (including CPAP) identified by procedure codes and diagnosis codes. If a surgical procedure was performed, the ventilatory support was required to be performed either 1 day before or 5 days after surgeries to be able to meet this criterion	Minor; score = 2
*Imaging* We included patients that received computed tomography (CT) or magnetic resonance imaging (MRI) with completed encounter order status or preliminary/final results available from radiological exams	Minor; score = 1
*Genetic diagnostic tests* We included patients who received genetic diagnostic tests such as gene sequencing or array comparative genomic hybridization regardless of test results. The records of genetic diagnostic tests were retrieved from procedure codes and labs	Minor; score = 1
*Metabolic diagnostic tests* We included patients who received metabolic tests such as a plasma amino acids panel or a urine organic acids panel, regardless of test results. The records of metabolic diagnostic tests were retrieved from procedure codes and labs	Minor; score = 1
*In-hospital death* Death information was retrieved from discharge location/disposition (expired, to funeral home/morgue or organ harvest) from encounter records	Major; score = 3
*Developmental delay* Patients with developmental delay were identified by either a specialist visit with a developmental pediatrician or at least two occurrences of related ICD codes (Supplemental Table 2B)	Minor; score = 1
*Diagnosis codes corresponding to metabolic diseases with* ≥ *2 encounters* We included patients with ICD codes for metabolic diseases (Supplemental Table 2C)	Major; score = 3
*Heart surgeries* Newborns that received heart surgeries were identified by encounters related to cardiothoracic surgeries or cardiothoracic intensive care unit (CTICU)	Major; score = 3

The cut-offs and scores for *PheIndex* were chosen and calibrated to mimic commonly observed healthcare utilization patterns among children presenting with illness with an increased risk for genetic disorders. Specifically, the distribution for various cutoffs per criterion was calculated, and the most reasonable cutoff was chosen based on the distribution in the population with reference to clinical relevance in identifying children with rare genetic disorders (see Supplemental Table S3A-D). Based on the severity of illness reflected by each rule, we classified 5 out of these 13 criteria as “major” and the remaining 8 as “minor”, as well as a score for each criterion scaled from 1 to 3, to account for the severity of illness in a clinical setting. A score of 3 indicates a criterion correlating with more severe illness, whereas a score of 1 reflects less severe illness. *PheIndex* combines these criteria in two different ways: (1) “*PheIndex Score*”, a score indicating the severity of illness with a possible range between 0 and 24 generated by the sum of the score(s) associated with the criteria met by a child; and (2) “*PheIndex Classification*”, a binary classification of those who present with illness with an increased risk for genetic disorders (*PheIndex Classification* positive) if the following conditions are met: (a) ≥2 major criteria, (b) ≥1 major criteria and ≥1 minor criteria, (c) ≥5 minor criteria, or (d) deceased patient; or those who do not present illness with increased risk for genetic disorders (*PheIndex Classification* negative).

### Chart review verification of the PheIndex digital phenotyping

To assess the accuracy of our *PheIndex* digital phenotyping algorithm, manual chart review was conducted in a blinded fashion for the validation of the 13 criteria listed in Table 4. Since we used structured, deidentified data for developing the digital phenotyping algorithm, full clinical information may not be present, particularly for specific clinical features noted in free text format in clinical notes. Therefore, blinded chart review by physicians is necessary. In the chart review process, a pediatrician examined all the clinical data for each patient including medical history such as birth history; all encounters including corresponding notes for outpatient, emergency department, and inpatient care; lab orders; imaging studies; and death records within the hospital medical records system to ascertain the presence of clinical criteria that comprise our *PheIndex* digital phenotyping. We selected 200 charts consisting of children who were *PheIndex Classification* positive (*N* = 100) and *PheIndex Classification* negative (*N* = 100). We ensured that the 100 children who were negative covered scores from 0 to 6 (inclusive), and from 3 to 21 for 100 children who were positive, based on the distribution of the *PheIndex Score* (see Fig. 1D in main text). Available records for this review were from encounters dated 01/01/2005 to 06/30/2020. All criteria determinations were based on available medical records up until three years of age. Chart selection covered each rule that we used to identify the phenotype to ensure representation including gestational age, NICU stay, emergency room visits, hospitalizations and duration of hospitalizations, subspecialty visits/consultations, presence of gastrostomy tube, presence of tracheostomy tube or utilization of mechanical ventilation in the absence of surgery, CT or MRI imaging studies, metabolic testing, genetic testing, metabolic disease diagnosis, developmental delay, prior cardiac surgery, and death.

The review by the pediatrician had two steps: 1) validate the accuracy of the values assigned to each of the 13 criteria for each patient; and 2) summarize diagnostic information from the patient charts. The pediatrician had access to additional delivery notes, progress notes, admission/discharge summaries, and imaging notes. Information on diagnoses available in the notes documented by the pediatrician was then used by a clinical geneticist to decide whether the child presented with illness with an increased risk for genetic disorders. The possible categories of determination were: 1) “Definitively/possibly has genetic disorder diagnosis”, 2) “Does not have a genetic disorder”, 3) “Unknown, insufficient information to make determination on whether a genetic disorder was related with illness.” Both definitively has a genetic disorder diagnosis and possibly has one were grouped together as ‘definitively’ included children with a positive test report, while ‘possibly’ included children who have not yet undergone genetic testing or lack definitive confirmation through such testing.

### Statistical analysis

We note that hospital utilization patterns are known to vary between pre-term and full-term infants, since pre-term infants often have more clinical needs and prolonged NICU stays. To assess this, for each group we computed the similarity between all pairs of *PheIndex* criteria using the Jaccard index: 13 for full-term and 12 for pre-term newborns (prolonged NICU stay was not a included as a criterion as pre-term newborns stay in NICU for being pre-term and not necessarily related to a rare disorder diagnosis). We described continuous variables as their median and quantile range, and categorical variables as a number and percentage. We performed statistical tests by ANOVA or two sample t-test for continuous variables and Chi-square test for categorical variables, respectively.

We performed all analyses using R (version 3.6.1) and Python (version 3.7). We considered p < 0.05 as statistically significant.

### Supplementary Information

**Supplementary Material 1.**

**Supplementary Material 2.**

## Data Availability

The clinical data in the current study were used under license from the Mount Sinai Data Warehouse. As a result, this dataset is not publicly available (https://labs.icahn.mssm.edu/msdw/data-use-agreement/). Qualified researchers affiliated with the Mount Sinai Health System may apply for access to these data through the Mount Sinai Health System Institutional Review Board.
