# Supplementary material for: An algorithm to identify patients aged 0–3 with rare genetic disorders
Source: Orphanet J Rare Dis. 2024 May 2;19:183. doi: 10.1186/s13023-024-03188-9 (PMC11064409; doi:10.1186/s13023-024-03188-9)
Supplement: Supplementary file 1 — Supplementary Material 1. [file 13023_2024_3188_MOESM1_ESM.pdf]

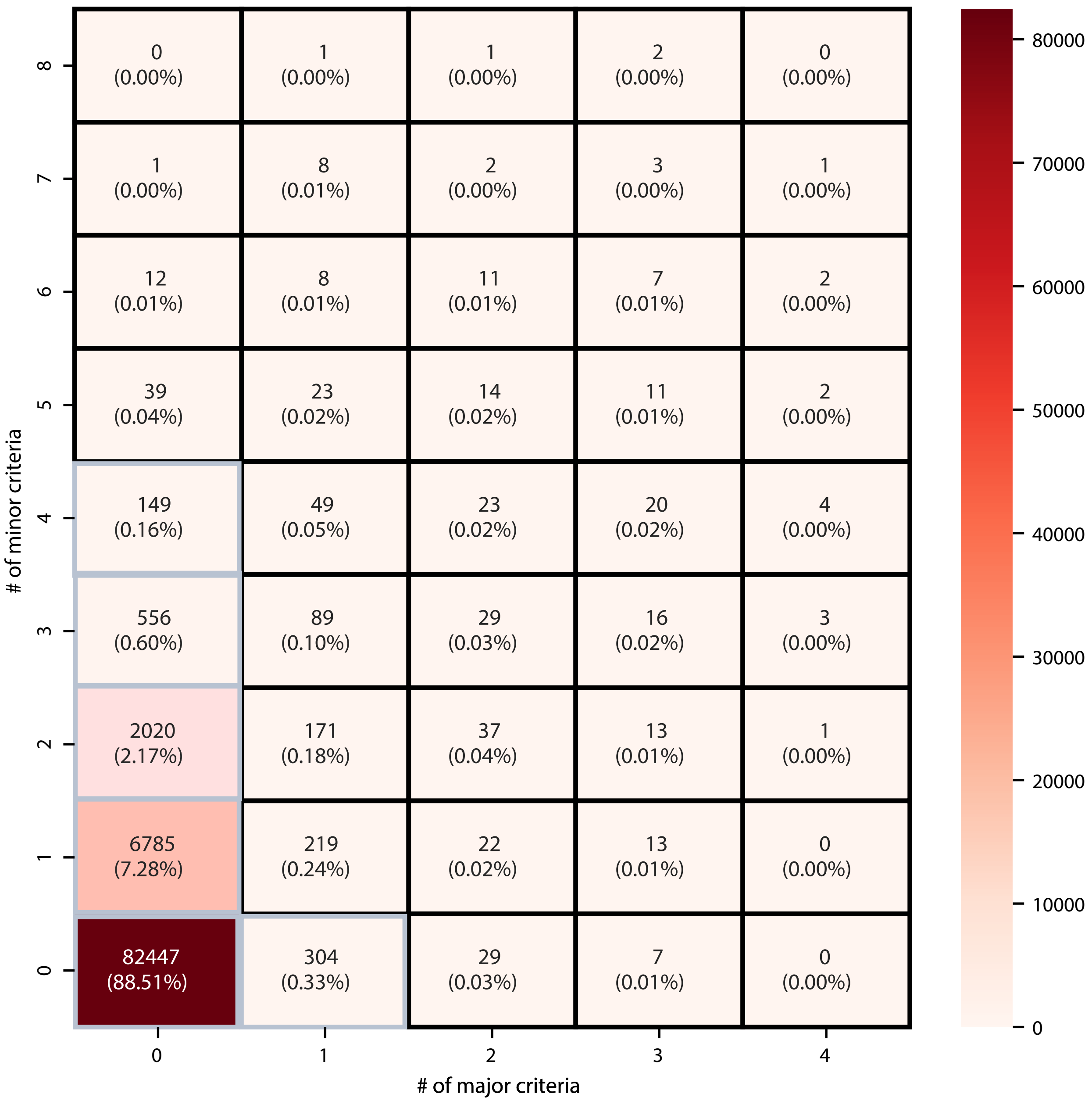

Figure S1: Number of children with each number of major and minor criteria from PheIndex digital phenotype. Square with grey borders indicate major/minor criteria combination that result in a "positive" label from the digital phenotype.
