# Supplementary material for: An algorithm to identify patients aged 0–3 with rare genetic disorders
Source: Orphanet J Rare Dis. 2024 May 2;19:183. doi: 10.1186/s13023-024-03188-9 (PMC11064409; doi:10.1186/s13023-024-03188-9)
Supplement: Supplementary file 2 — Supplementary Material 2. [file 13023_2024_3188_MOESM2_ESM.docx]

**Supplementary Table S1: Number of children passing each individual *PheIndex* criteria.**

| **PheIndex Criteria** | **n (%)** |
| --- | --- |
| multiple ER visits | 3919 (4.2) |
| developmental delay | 3159 (3.4) |
| multiple specialists | 3091 (3.3) |
| respiratory support | 2838 (3.0) |
| imaging | 1113 (1.2) |
| genetic tests | 704 (0.8) |
| prolonged in-patient stays | 500 (0.5) |
| metabolic tests | 448 (0.5) |
| death | 371 (0.4) |
| heart surgeries | 304 (0.3) |
| prolonged NICU stay | 279 (0.3) |
| feeding support | 132 (0.1) |
| metabolic ICD codes | 82 (0.1) |

**Supplemental Table S2A: Gastronomy ICD codes**

| V44.1 (ICD9) | Gastrostomy status |
| --- | --- |
| 779.31 (ICD9) | Feeding problems in newborn |
| Z93.1 (ICD10) | Gastrostomy status |
| P92.9 (ICD10) | Feeding problem of newborn, unspecified |

**Supplemental Table S2B: Developmental delay ICD codes**

| 315.3 (ICD9) | Expressive language disorder |
| --- | --- |
| 315.31 (ICD9) | Expressive language disorder |
| 315.32 (ICD9) | Mixed receptive-expressive language disorder |
| 315.39 (ICD9) | Other developmental speech or language disorder |
| 315.4 (ICD9) | Developmental coordination disorder |
| 315.8 (ICD9) | Other specified delays in development |
| 783.40 (ICD9) | Lack of normal physiological development |
| V40.0 (ICD9) | Mental and behavioral problems with learning |
| R62.50 (ICD10) | Developmental delay |
| F80.1 (ICD10) | Expressive language delay |
| F80.2 (ICD10) | Receptive expressive language disorder |
| F80.9 (ICD10) | Speech developmental delay |
| F81.9 (ICD10) | Cognitive developmental delay |
| F82 (ICD10) | Developmental delay of gross and fine motor function |
| F88 (ICD10) | Global developmental delay |
| Z87.898 (ICD10) | History of developmental delay |

**Supplemental Table S2C: Diagnosis codes corresponding to metabolic diseases**

| E70* (excl. E70.331: Hermansky-Pudlak syndrome) | Disorders of aromatic amino-acid metabolism |
| --- | --- |
| E71* | Disorders of branched-chain amino-acid metabolism and fatty-acid metabolism |
| E72* | Other disorders of amino-acid metabolism |
| E74* | Other disorders of carbohydrate metabolism |
| E75* | Disorders of sphingolipid metabolism and other lipid storage disorders |
| E76* | Disorders of glycosaminoglycan metabolism |
| E78* | Disorders of lipoprotein metabolism and other lipidemias |
| D81.810 | Biotinidase deficiency |

**Supplemental Table S3A: Distribution of NICU length of stay in the study cohort among full-term births**

| Length of stay | % |
| --- | --- |
| 0 | 5.955 |
| 1 | 5.796 |
| 2 | 4.900 |
| 3 | 3.623 |
| 4 | 2.839 |
| 5 | 2.317 |
| 6 | 1.928 |
| 7 | 1.662 |
| 8 | 1.444 |
| 9 | 1.301 |
| 10 | 1.165 |
| 11 | 1.038 |
| 12 | 0.957 |
| 13 | 0.898 |
| 14 | 0.849 |
| 15 | 0.792 |
| 16 | 0.733 |
| 17 | 0.697 |
| 18 | 0.674 |
| 19 | 0.639 |

**Supplemental Table S3B: Distribution of number of inpatient stays with a duration ≥2 days in the study cohort among preterm and full-term births**

| Number of inpatient stays | Preterm % | Full-term % |
| --- | --- | --- |
| 1 | 18.623 | 3.917 |
| 2 | 2.275 | 0.554 |
| 3 | 0.988 | 0.207 |
| 4 | 0.453 | 0.116 |
| 5 | 0.175 | 0.065 |
| 6 | 0.113 | 0.047 |
| 7 | 0.082 | 0.031 |
| 8 | 0.072 | 0.023 |
| 9 | 0.062 | 0.015 |
| 10 | 0.041 | 0.013 |
| 11 | 0.041 | 0.012 |
| 12 | 0.021 | 0.008 |
| 13 | 0.010 | 0.006 |
| 14 | 0 | 0.006 |
| 15 | 0 | 0.005 |
| 16 | 0 | 0.004 |
| 22 | 0 | 0.002 |
| 24 | 0 | 0.001 |

**Supplemental Table S3C: Distribution of number of emergency room (ER) visits in the study cohort among preterm and full-term births**

| **Number of ER visits** | **Preterm %** | **Full-term %** |
| --- | --- | --- |
| 0 | 100 | 100 |
| 1 | 21.876 | 16.328 |
| 2 | 14.865 | 10.655 |
| 3 | 10.366 | 7.306 |
| 4 | 7.927 | 5.514 |
| 5 | 6.156 | 4.263 |
| 6 | 4.982 | 3.295 |
| 7 | 4.015 | 2.572 |
| 8 | 3.140 | 2.059 |
| 9 | 2.574 | 1.651 |

**Supplemental Table S3D: Distribution of number of specialist visits in the study cohort among preterm and full-term births**

| Number of specialist visits | Preterm % | Full-term % |
| --- | --- | --- |
| 1 | 38.511 | 19.993 |
| 2 | 22.174 | 7.453 |
| 3 | 13.197 | 3.172 |
| 4 | 7.988 | 1.471 |
| 5 | 4.787 | 0.696 |
| 6 | 2.965 | 0.363 |
| 7 | 1.627 | 0.196 |
| 8 | 0.988 | 0.112 |
| 9 | 0.566 | 0.062 |
| 10 | 0.381 | 0.043 |
| 11 | 0.165 | 0.031 |
| 12 | 0.082 | 0.020 |
| 13 | 0.010 | 0.011 |
| 14 | 0.010 | 0.004 |
| 15 | 0.010 | 0.001 |
